# A case-control study on the role of anatomical factors in nontraumatic lateral osteochondral lesion development of the talus

**DOI:** 10.1097/MD.0000000000043586

**Published:** 2025-07-25

**Authors:** Eda Cingoz, Mehmet Cingoz, Rana Gunoz Comert, Memduh Dursun

**Affiliations:** a Radiology Department, Bagcilar Training and Research Hospital, Istanbul, Turkey; b Radiology Department, Basaksehir Cam and Sakura City Hospital, Istanbul, Turkey; c Radiology Department, Istanbul Medical Faculty, Istanbul University, Istanbul, Turkey.

**Keywords:** lateral, MRI, osteochondral lesion, talus

## Abstract

Lateral osteochondral lesions of the talus (OLT) are a notable cause of chronic ankle pain, particularly in cases without a history of trauma. However, their anatomical risk factors remain understudied compared to medial OLTs. This study aimed to identify anatomical factors associated with the development of nontraumatic lateral OLT using magnetic resonance imaging (MRI)-based morphometric measurements. MRI scans from 62 ankles were examined in this retrospective study, comprising 31 patients with lateral OLT and 31 healthy controls matched by age, sex, and side. The following parameters were measured on the MR images: the anterior opening angle of the talus (AOT), the angle between the tibial axis and medial malleolus (TMM), the angle of the tibial plafond to the malleolus, the angle between the anterior and posterior tibiofibular ligaments (ATFL–PTFL angle), the length of the distal tibial articular surface (TAS), the length of the trochlea tali arc (TAL), the ratio of TAS to TAL (TAS/TAL), and the depth of the incisura fibularis (IncDep). AOT, IncDep, ATFL–PTFL angle, TMM, TAL, and TAS/TAL demonstrated significant differences between the 2 groups (*P* < .05). The threshold values were as follows: 13.5° (area under the curve [AUC] 0.807) for AOT, 80.5° (AUC 0.767) for ATFL–PTFL angle, 16.5° (AUC 0.920) for TMM, and 0.80 (AUC 0.704) for TAS/TAL. Multivariate logistic regression analysis indicated an odds ratio (OR) = 17.805 for AOT ≥ 13.5°, OR = 19.887 for ATFL–PTFL angle > 80.5°, OR = 27.576 for TMM > 1.5° and OR = 4.680 for TAS/TAL ≤ 0.80. These findings suggest that specific anatomical parameters identifiable on MRI, particularly increased AOT, TMM, and ATFL–PTFL angle, are significantly associated with the development of nontraumatic lateral OLT. These parameters may serve as useful imaging biomarkers for clinical risk assessment.

## 1. Introduction

Osteochondral lesions of the talus (OLTs) result from damage to the talar articular cartilage and the underlying subchondral bone. Osteochondral lesions were initially characterized by König in 1887 for the knee and subsequently delineated within the talus by Kappis et al in 1922.^[1–3]^ OLTs are considered to be a prevalent etiology for persistent ankle discomfort and functional impairment. Inadequate management may predispose to precocious cartilaginous degeneration and subsequent osteoarthritic sequelae. Although many cases of lateral OLTs result from traumatic events like ankle sprains or fractures, a significant number also emerge from nontraumatic origins, including idiopathic or hereditary factors.^[4–6]^

Medial OLTs are generally more prevalent and are linked to worse prognosis due to largely unknown causes, whereas lateral OLTs are less frequent but tend to have a more favorable prognosis.^[7,8]^ Approximately half of the individuals diagnosed with lateral OLT have a documented history of traumatic events while the other half are of nontraumatic origin.^[9,10]^ Due to the higher incidence of medial OLT and the ambiguity surrounding its causation, there has been a considerable focus in the literature on investigating its anatomical risk factors.^[11,12]^ A similar focus has evaded lateral OLT cases as they are mainly attributed to trauma and occur less frequently. Although a large proportion of the literature addressing OLT focuses on medial-sided pathology, studies specifically investigating lateral OLT from an anatomical perspective are scarce. Most anatomical studies either group medial and lateral lesions together or exclude lateral OLT entirely due to its presumed traumatic etiology. Nonetheless, recent clinical interest in lateral lesions has grown, particularly regarding treatment outcomes. Clinical and radiologic outcomes have been reported following autologous osteochondral transplantation for lateral OLTs, and the transplantation and iliac crest-based osteoperiostic grafting technique has shown promising results for large lateral lesions.^[13,14]^ Despite these therapeutic advancements, there remains a paucity of anatomical studies that investigate potential morphological risk factors unique to lateral OLT.

The aim of the current study was to investigate morphological characteristics of the ankle that may increase the risk for the development of lateral OLT. Utilizing magnetic resonance imaging (MRI), the current study also included an evaluation of previously examined factors associated with medial OLT and the investigation of novel anatomical determinants that may be specific to lateral OLT.

## 2. Methods

### 2.1. Study design and population

Ankle MRI images generated between January 2020 and December 2023 were reviewed in this retrospective study. The necessity for informed consent was waived due to the retrospective design of the study, and the research protocol received approval from the institutional ethics committee (reference number: 2023/1890). A board-certified radiologist specializing in musculoskeletal imaging with 7 years of experience conducted a thorough assessment of the ankle MR images. A total of 62 ankle MRIs were analyzed, comprising 31 cases with lateral-sided OLT and 31 controls with normal ankle MRI findings, both matched for age, gender, and the side. Individuals with a history of significant trauma, foot anomalies, prior ankle surgeries, damage to the anterior or posterior talofibular ligaments, and pronounced osteoarthritic alterations were excluded.

### 2.2. MRI acquisition and image analysis

**Figure 1.**
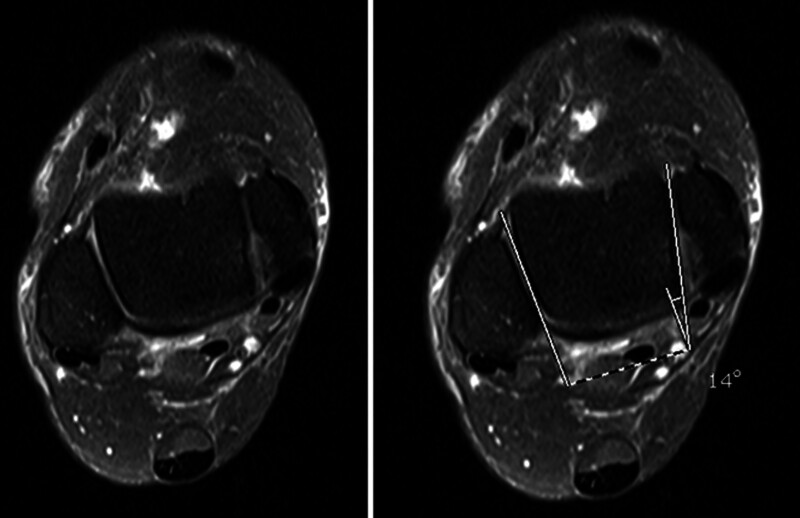
The anterior opening angle of the talus (AOT) was determined in axial STIR sequences by delineating 2 lines along the medial and lateral facets of the talar dome, followed by the measurement of the angle formed between these lines to ascertain the AOT.

**Figure 2.**
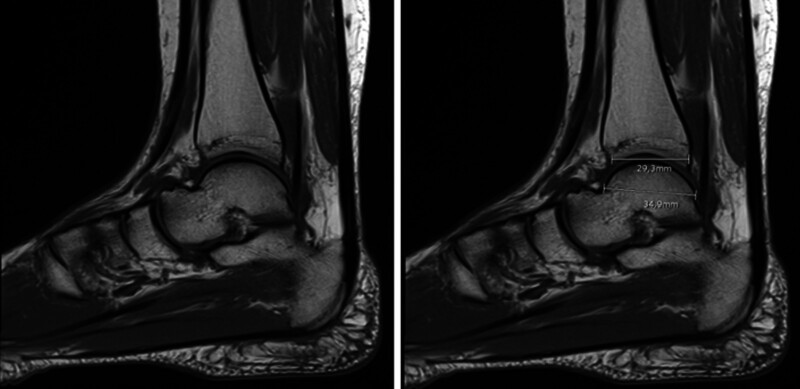
The span of the distal tibial articular surface (TAS) in the sagittal plane and the extent of the trochlea tali arc (TAL) were quantified from sagittal T1-weighted images. TAS was assessed by measuring the most anterior to the most posterior point on the distal tibial articular surface, whereas TAL was determined by the distance between the farthest anterior and posterior points of the trochlea tali. The TAS/TAL ratio was generated to standardize these dimensions.

**Figure 3.**
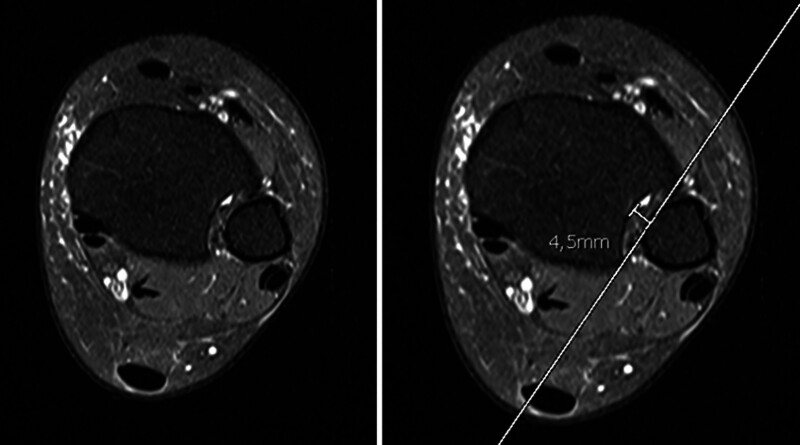
The assessment of the incisura fibularis depth (IncDep) entailed the delineation of a line spanning the anterior and posterior edges of the incisura fibularis on axial STIR images. IncDep was determined by measuring the perpendicular distance from this line to the deepest part of the incisura.

**Figure 4.**
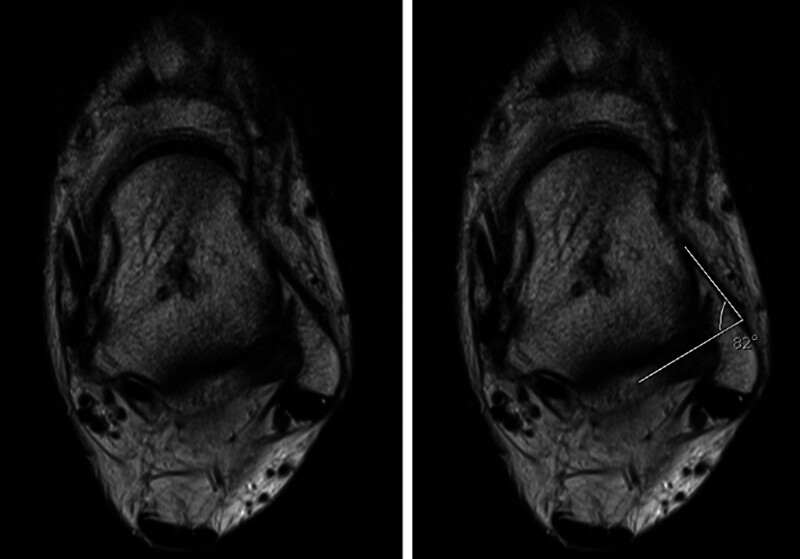
A specific imaging plane was defined from axial T2-weighted images where the anterior talofibular ligament (ATFL) and the posterior talofibular ligament (PTFL) were simultaneously visible. Within this plane, 2 lines were drawn: one parallel to the ATFL and the other parallel to the PTFL. The angle formed by these lines, known as the ATFL–PTFL angle, was measured.

**Figure 5.**
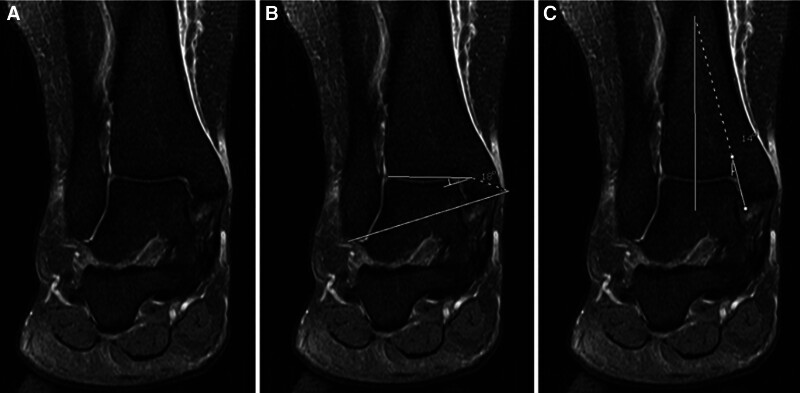
(A) The plafond–malleolar angle (PMA) was determined by the angle between the joint orientation line of the distal tibia articular surface and the transmalleolar axis in coronal STIR images, (B) The PMA was drawn by connecting the distal tip of the medial malleolus to the distal tip of the lateral malleolus. This illustrated the alignment between the tibial plafond and the transmalleolar axis. (C) The tibial axis-medial malleolus angle (TMM) was measured as the angle between the longitudinal axis of the tibia and a line parallel to the joint surface of the medial malleolus.

**Figure 6.**
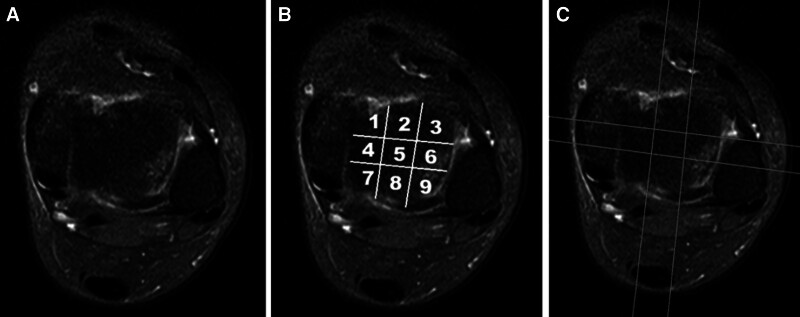
(A, B) The lesions in osteochondral lesions of the talar dome (OLT) were localized with a 9-grid scoring system proposed by Elias et al. The affected areas within this grid, as well as the total number of involved regions, were recorded. (C) Stage 2a lateral OLT were confined to areas 6 and 9, leading to a count of 2 impacted regions.

Several crucial anatomical parameters were evaluated using the ankle MRI scans. These parameters included the anterior opening angle of the talus (AOT), the length of the distal tibial articular surface (TAS), the length of the trochlea tali arc (TAL), the ratio of the TAS to TAL (TAS/TAL), the depth of the incisura fibularis (IncDep), the angle between the anterior talofibular ligament and the posterior talofibular ligament (ATFL–PTFL angle), and the plafond–malleolar angle (PMA), along with the angle formed by the tibial axis and the medial malleolus (TMM) (Figs. 1–5, respectively). These parameters were selected as they are critical for understanding the anatomical structure of the ankle and due to their potential influence on increasing articular contact stress.^[12,15,16]^ Unlike previous investigations, the current study not only introduced the PMA as a novel parameter but also expanded the analysis to include the evaluation of ligamentous structures, notably the ATFL–PTFL angle. This approach allowed for a detailed comparison between patients with lateral OLT and the control group (Figs. 4 and 5) and helped enhance the understanding of ankle biomechanics. The current research utilized the 9-grid scoring system by Elias et al to accurately locate lesions in patients with lateral OLT. The affected zones and the total lesion counts were documented (Fig. 6).^[17]^ The Hepple methodology was used for the classification of OCL, where the measurement of the lesion was ascertained in the dimension where its maximum length was observed. In the categorizations 2A, 3, and 4, the dimension of the OCL encompassed the osseous fragment, but did not account for the surrounding zone consisting of reactive edema. Additionally, accessory ossicles around the ankle were carefully identified and each type was classified when encountered.

MR imaging of the ankle was carried out using 1.5 Tesla systems from 2 different manufacturers, (MAGNETOM Amira, Siemens Healthcare, Germany, and the Ingenia, Philips Healthcare, Best, The Netherlands). Dedicated 16-channel ankle coils were employed for the examinations, which were carried out with the patient in a supine position and the ankle positioned in neutral alignment, with the toes pointing straight up, following the protocols outlined in Table 1. The coronal plane was demarcated in alignment parallel to the longitudinal axis of the distal tibia, whereas the sagittal plane was designated as orthogonal to the coronal plane. Concurrently, the axial plane was characterized as orthogonal to both the coronal and sagittal planes, and was configured in parallel orientation with respect to the talus dome.

**Table 1:** Magnetic resonance imaging protocols for ankle and their characteristics.

Imaging orientation	Imaging technique	Repetition time (TR) in ms	Echo time (TE) in ms	Slice thickness (ST) in mm	Field of view (FOV) in mm
Sagittal	T1-weighted TSE	609	12	3.5	165
Sagittal	STIR TSE	3090	42	3.5	170
Coronal	PD-weighted TSE	2560	34	3	170
Axial	T2-weighted TSE	3350	88	3.5	140
Axial	PD-weighted TSE	4300	66	3.5	160

FOV = field of view, PDW = proton density weighted, ST = thickness of slice, STIR = short-tau inversion recovery, TE = echo time, TR = repetition time, TSE = turbo spin-echo.

### 2.3. Statistical analysis

Statistical analysis was carried out using SPSS for Windows version 18.0 (SPSS Inc., Chicago, IL) software package. The normality of the data distribution was examined by using both visual (histograms and probability plots) and analytical methods (Shapiro–Wilk test). For the evaluation of numerical data, mean, standard deviation, and median (1st quartile–3rd quartile) values were used; for summarizing categorical data, frequency distributions and percentages were employed. The analysis of numerical data that conformed to a normal distribution was conducted using the independent samples *t* test, while numerical data that did not follow a normal distribution were analyzed using the Mann–Whitney *U* test. Comparison of categorical data was carried out using Pearson chi-square test. The suitability of the measured parameters in predicting OLT for diagnostic purposes was examined using the receiver operating characteristics curve. Where significant threshold values were present, the sensitivity, specificity, positive predictive value, and negative predictive value of these thresholds were calculated. Independent factors associated with OLT using the identified potential variables were examined using univariate and multivariate logistic regression analysis. Covariates to be included in the multivariate logistic regression model were initially evaluated through univariate analyses. Variables demonstrating statistical significance at a level of *P* < .10 in the univariate analysis were considered candidates for inclusion in the multivariate model. In addition, clinically important covariates such as age and sex, which have been reported in the literature to have a significant impact, were also incorporated into the model. This approach allowed for the control of variables that may be relevant from both statistical and clinical standpoints. The goodness-of-fit of the multivariate logistic regression model was assessed using the Hosmer–Lemeshow test (*P* = .527), indicating a good fit to the data. The explanatory power of the model was evaluated using the Nagelkerke R square, which was found to be 0.750. This value indicates that the model accounts for approximately 75% of the variance in the dependent variable, namely the presence of OCD. *P* < .05 was considered to be statistically significant.

## 3. Results

### 3.1. Clinical and morphological findings

The participants included 34 males (54.8%) and 28 females (45.2%), with an age range of 24 to 50 years and a mean age of 37.90 ± 14.28 years. The OLT stage in the patients was identified as 2a in 71%, 5 in 22.6%, and 3 in 6.5% of the patients. The most frequently affected area in lateral OLT was zone 6, followed by zone 9 (Table 2). Individuals with OLT had significantly higher measurements of AOT, IncDep, ATFL–PTFL angle, TMM, and TAL compared to the healthy individuals (*P* < .05). The TAS/TAL ratio was significantly lower in patients with OLT compared to the healthy subjects (*P* = .006). Accessory bones were present in 48.4% of patients with OLT and 22.6% of the healthy individuals (*P* = .031). PMA, TAS, the presence of the os trigonum, and accessory navicular bone were similar between the 2 groups (*P* > .05, Table 3).

**Table 2:** Features of osteochondral lesions of the talar dome.

Features		OLT group (n = 31)
		n (%)
Side	Right	9 (29.0)
	Left	22 (71.0)
Stage	Stage 2a	22 (71.0)
	Stage 3	2 (6.5)
	Stage 5	7 (22.6)
The number of OLT locations	1 Location	20 (64.5)
	2 Locations	7 (22.6)
	3 Locations	3 (9.7)
	4 Locations	1 (3.2)
The location of OLT	Zone 3	7 (22.6)
	Zone 5	2 (6.5)
	Zone 6	20 (64.5)
	Zone 8	1 (3.2)
	Zone 9	17 (54.8)
Size of OLT (mm)	Mean ± SD	8.56 ± 4.79
	Median (1st–3rd quartile)	7.40 (5.00–10.00)

OLT = osteochondral lesions of the talar dome.

**Table 3:** Comparison of ankle measurement values between the patients and healthy individuals.

Variables	Groups		*P*
	OLT (n = 31)	Healthy (n = 31)	
	Mean ± SD Median (1st–3rd quartile)	Mean ± SD Median (1st–3rd quartile)	
AOT	15.54 ± 3.36 16.00 (14.00–19.00)	12.48 ± 1.58 13.00 (12.00–14.00)	**<.001** *
IncDep	4.00 ± 1.18 4.10 (3.00–4.80)	3.32 ± 0.88 3.40 (2.80–3.90)	**.013** *
ATFL–PTFL angle	81.96 ± 6.31 84.00 (76.00–86.00)	75.80 ± 5.59 77.00 (71.00–80.00)	**<.001** *
PMA	15.67 ± 2.76 16.00 (14.00–17.00)	16.29 ± 2.80 16.00 (15.00–19.00)	.390*
TMM	19.22 ± 3.84 18.00 (17.00–22.00)	13.51 ± 2.27 14.00 (12.00–15.00)	**<.001** *
TAS/TAL	0.78 ± 0.05 0.79 (0.74–0.82)	0.83 ± 0.06 0.82 (0.79–0.84)	**.006** †
TAS	28.56 ± 3.21 29.20 (26.80–30.30)	28.41 ± 2.51 28.20 (31.30–37.10)	.847*
TAL	36.41 ± 4.58 36.80 (32.40–39.80)	34.33 ± 3.35 34.50 (31.30–37.10)	**.045** *

	n (%)	n (%)	
Accessory bone			
Present	15 (48.4)	7 (22.6)	**.031** ‡
Absent	16 (51.6)	24 (77.4)	
Os trigonum			
Present	9 (29.0)	3 (9,7)	.054‡
Absent	22 (71.0)	28 (90.3)	
Accessory navicular bone			
Present	7 (22.6)	3 (9.7)	.167‡
Absent	24 (77.4)	28 (90.3)	

Bold values indicate statistical significance at *P* < .05. AOT = angle of the talus, ATFL = anterior talofibular ligament, AUC = area under the curve, OLT = osteochondral lesions of the talus, PMA = plafond–malleolar angle, PTFL = posterior talofibular ligament, TAL = the length of the trochlea tali arc, TAS = tibial articular surface, TMM = the angle between the tibial axis and medial malleolus.

* Independent samples *t* test.

† Mann–Whitney *U* test.

‡ Pearson chi-square test.

### 3.2. Diagnostic analysis and predictive modeling

**Figure 7.**
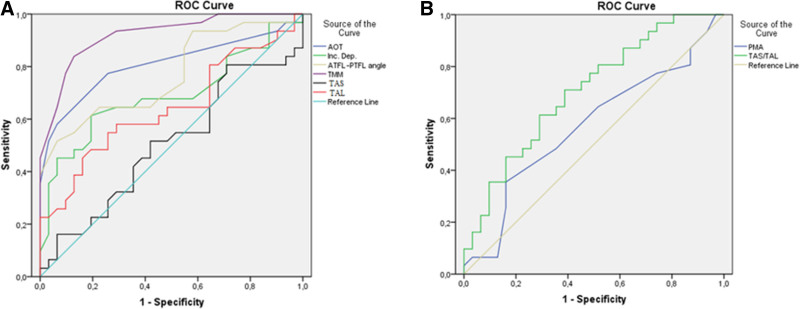
ROC curves for AOT, IncDep, ATFL–PTFL angle, TMM, TAS, TAL, PMA, TAS/TAL ratio. (A) Higher values were associated with the presence of OLT; (B) Lower values were more indicative of OLT. ROC = receiver operating characteristics.

The area under the curve (AUC) and cutoff values for significant parameters are as follows: AOT (AUC = 0.807, cutoff = 13.5°), ATFL–PTFL angle (AUC = 0.767, cutoff = 80.5°), TMM (AUC = 0.920, cutoff = 16.5°), and TAS/TAL ratio (AUC = 0.704, cutoff = 0.8) (Table 4 and Fig. 7). A regression model incorporating these threshold values was established to assess the likelihood of presenting with lateral OLT. Individuals with an AOT value of 13.5° or greater had a 17.805-fold increase, those with an ATFL–PTFL angle of 80.5° or greater had a 19.887-fold increase, and those with a TMM of 16.5° or greater had a 27.576-fold increase in the likelihood of presenting with OLT (Table 5). AOT, the ATFL–PTFL angle, TMM, and the TAS/TAL ratio were identified as significant factors in the univariate analysis. However, the significance of TAS/TAL ratio was not retained in the multivariate analysis.

**Table 4:** Results of the ROC analysis for the measured parameters.

Parameters	AUC (95% CI)	*P*	Cutoff	Sensitivity (%)	Specificity (%)	PPV (%)	NPV (%)
AOT	0.807 (0.693–0.922)	**<.001**	13.5	77.4	74.2	75.0	76.7
IncDep	0.689 (0.553–0.826)	.010					
ATFL–PTFL angle	0.767 (0.648–0.886)	**<.001**	80.5	64.5	77.4	74.1	68.6
PMA	0.569 (0.424–0.714)	.349					
TMM	0.920 (0.854–0.987)	**<.001**	16.5	77.4	90.3	88.9	80.0
TAS/TAL	0.704 (0.576–0.833)	**.006**	0.8	71.0	61.3	64.7	67.9
TAS	0.503 (0.356–0.649)	.972					
TAL	0.642 (0.502–0.781)	.056					

Bold values indicate statistical significance at *P* < .05. AOT = angle of the talus, ATFL = anterior talofibular ligament, AUC = area under the curve, CI = confidence interval, NPV = negative predictive value, PMA = plafond–malleolar angle, PPV = positive predictive value, PTFL = posterior talofibular ligament, ROC = receiver operating characteristics, TAL = the length of the trochlea tali arc, TAS = tibial articular surface, TMM = the angle between the tibial axis and medial malleolus.

**Table 5:** Results of the univariate and multivariate logistic regression analyses conducted to identify independent factors on osteochondral lesion.

Variables	Univariate analysis		Multivariate analysis	
	OR (95% CI)	*P*	OR (95% CI)	*P*
AOT ≥ 13.50 (Ref < 13.50)	9.857 (3.076–31.585)	**<.001**	17.805 (2.146–147.749)	**.008**
ATFL–PTFL ≥ 80.50 (Ref < 80.50)	6.234 (2.038–19.069)	**.001**	19.887 (2.055–192.437)	**.010**
TMM ≥ 16.50 (Ref < 16.50)	32.000 (7.445–137.551)	**<.001**	27.576 (0.3719–204.476)	**.001**
TAS/TAL ≤ 0.8047 (Ref > 0.8047)	3.870 (1.341–11.172)	**.012**	4.680 (0.544–40.233)	.160

Hosmer–Lemeshow: 0.527, Nagelkerke R square: 0.750. Bold values indicate statistical significance at *P* < .05. AOT = angle of the talus, ATFL = anterior talofibular ligament, CI = confidence interval, OR = odds ratio, PTFL = posterior talofibular ligament, TAL = the length of the trochlea tali arc, TAS = length of tibial articular surface, TMM = the angle between the tibial axis and medial malleolus.

## 4. Discussion

The angle delineated by the medial and lateral aspects of the trochlear surface of the talus characterizes the AOT of the talus. This parameter is pivotal in the dynamics of ankle biomechanics. Notably, the talus exhibits greater width anteriorly than posteriorly, contributing to diminished stability during movements involving plantar flexion, as it occupies the ankle mortise to a lesser extent. An increased AOT implies a comparative reduction in the posterior dimensions of the talus, potentially compromising stability, especially in plantar flexion scenarios. This alteration may lead to an altered distribution of axial forces, thereby increasing the risk of OLT. A statistically significant association was established between the risk of presenting with lateral OLT and an AOT exceeding 13.5° in the current study.

Prior investigations have underscored the critical role of the positioning of the fibula in accentuating the axial malleolar index within the horizontal axis. This was reported to serve as the principal anatomical characteristic in individuals with ankle instability that could potentially increase their susceptibility to OLT.^[18]^ Our theory posited that the depth of incisura fibularis might influence the overall configuration of the ankle; a deeper incisura fibularis may potentially cause a shift of the fibula towards a more medial direction, and consequently reposition the lateral malleolus. Such a shift in the positioning of the fibula could alter the configuration of the talar dome, leading to its reduced clearance, and an elevated risk of developing OLT. The evidence gathered from our study corroborates this hypothesis, as we have identified a significant association between high value of IncDep and a risk of OLT.

Previous research has indicated that an increased angle between the ATFL and PTFL may serve as an indirect MRI-based marker for the diagnosis of chronic injury to the ATFL.^[19]^ Following minor to moderate trauma, the ATFL is prone to deformation and dysfunction, while the PTFL often remains unaffected, leading to increased angular disparity between these ligaments. Zhang et al defined a threshold value for the ATFL–PTFL angle at 85.05° in a cohort of 120 individuals with chronic ankle instability in comparison with a control group of similar size.^[20]^ However, the potential link between the ATFL–PTFL angle and the incidence of OLT has not been reported yet. A higher ATFL–PTFL angle in patients with lateral OLT was detected in the current study, in comparison to a healthy control group, establishing a new threshold value exceeding 80.5° for the ATFL–PTFL angle. The current study is the first to establish a relationship between the ATFL–PTFL angle and the occurrence of lateral OLT.

An increase in TMM might contribute to enhanced talus mobility within the confines of the medial and lateral malleoli, which may subsequently lead to instability in the ankle region. Previous studies have shown that the TMM, quantified by anteroposterior radiographic imaging, was typically enlarged in patients who presented with more severe chondral damage.^[21]^ Moreover, a separate study has established an association between increased TMM and the occurrence of medial-sided OLT.^[12]^ Our research also supports such a significant association and has identified a threshold of 16.5° for TMM, thereby underlining the significance of the TMM in the risk of presenting lateral-sided OLT.

An elevated TAL was correlated with an increased incidence of lateral-sided OLT in the current study, supporting findings from previous studies that have suggested a similar correlation for medial-sided OLT. These data support the hypothesis that an enlarged TAL might act as an indicator for OLT.^[11,22]^ Univariate analyses indicated a significant correlation in the TAS/TAL ratio between the patient and control groups, suggesting an association of this ratio with the development of lateral OLT. This association may be ascribed to a reduced relative contact surface between the tibia and talus, leading to a concentration of biomechanical stress over a diminished area on the talar dome in patients with OLT as opposed to individuals without such lesions. This modification in the biomechanical dynamics of the ankle may render individuals more prone to the development of OLT. Although these findings suggest that the TAS/TAL ratio might play a pivotal role in the evaluation of lateral-sided OLT risk factors, this factor did not retain its significance in the multivariate analysis, underscoring the complexity of factors contributing to lateral OLT.

Patients with OLT showed a significant difference in the presence of accessory bones compared to the control group (*P* = .031). Thus, the presence of accessory bones in the foot may be considered as a risk factor for the development of lateral OLT; moreover, this relationship appeared to be independent of the accessory bone subtype. To our knowledge, this relationship has not been explored in the existing literature, making our study the first to highlight this potential risk factor. This study has several limitations, including its retrospective, single-center design, relatively small sample size, and the lack of evaluation of factors such as stature, body mass, and lower extremity alignment, all of which may affect the generalizability of the findings.

## 5. Conclusion

In conclusion, our study has identified several risk factors for the development of lateral OLT. These include AOT exceeding 13.5°, ATFL–PTFL angle >80.5°, TMM >16.5°, and the presence of accessory foot bones, regardless of subtype. These findings underscore the multifaceted nature of the development of lateral OLT and highlight key anatomical parameters that could inform clinical assessments.

## Author contributions

**Data curation:** Eda Cingoz, Mehmet Cingoz.

**Investigation:** Mehmet Cingoz, Rana Gunoz Comert.

**Supervision:** Memduh Dursun.

**Writing – original draft:** Eda Cingoz, Rana Gunoz Comert.

**Writing – review & editing:** Mehmet Cingoz, Memduh Dursun.
